# Supplementary material for: Stress among medical students: factor structure of the University Stress Scale among Italian students
Source: BMJ Open. 2020 Sep 1;10(9):e035255. doi: 10.1136/bmjopen-2019-035255 (PMC7467511; doi:10.1136/bmjopen-2019-035255)
Supplement: Author's manuscript [file bmjopen-2019-035255.draft_revisions.pdf]

BMJ Open is committed to open peer review. As part of this commitment we make the peer review history of every article we publish publicly available.

When an article is published we post the peer reviewers' comments and the authors' responses online. We also post the versions of the paper that were used during peer review. These are the versions that the peer review comments apply to.

The versions of the paper that follow are the versions that were submitted during the peer review process. They are not the versions of record or the final published versions. They should not be cited or distributed as the published version of this manuscript.

BMJ Open is an open access journal and the full, final, typeset and author-corrected version of record of the manuscript is available on our site with no access controls, subscription charges or pay-per-view fees (http://bmjopen.bmj.com).

If you have any questions on BMJ Open's open peer review process please email info.bmjopen@bmj.com

BMJ Open

Stress Among Medical Students: bifactor measurement model of the University Stress Scale

Journal:	*BMJ Open*
Manuscript ID	bmjopen-2019-035255
Article Type:	Original research
Date Submitted by the Author:	28-Oct-2019

Complete List of Authors:	Portoghese, Igor; University of Cagliari, Medical Sciences and Public Health<br>Porru, Fabio; Erasmus Medical Center, Public Health; University of Cagliari, Medical Sciences and Public Health<br>Galletta, Maura; Universita degli Studi di Cagliari, Medical Sciences and Public Health<br>Campagna, Marcello; University of Cagliari, Medical Sciences and Public Health<br>Burdorf, Alex; Erasmus Medical Center, Public Health

Keywords:	EDUCATION & TRAINING (see Medical Education & Training), PUBLIC HEALTH, PREVENTIVE MEDICINE

SCHOLARONE™
Manuscripts

I, the Submitting Author has the right to grant and does grant on behalf of all authors of the Work (as defined in the below author licence), an exclusive licence and/or a non-exclusive licence for contributions from authors who are: i) UK Crown employees; ii) where BMJ has agreed a CC-BY licence shall apply, and/or iii) in accordance with the terms applicable for US Federal Government officers or employees acting as part of their official duties; on a worldwide, perpetual, irrevocable, royalty-free basis to BMJ Publishing Group Ltd ("BMJ") its licensees and where the relevant Journal is co-owned by BMJ to the co-owners of the Journal, to publish the Work in this journal and any other BMJ products and to exploit all rights, as set out in our licence.

The Submitting Author accepts and understands that any supply made under these terms is made by BMJ to the Submitting Author unless you are acting as an employee on behalf of your employer or a postgraduate student of an affiliated institution which is paying any applicable article publishing charge ("APC") for Open Access articles. Where the Submitting Author wishes to make the Work available on an Open Access basis (and intends to pay the relevant APC), the terms of reuse of such Open Access shall be governed by a Creative Commons licence – details of these licences and which Creative Commons licence will apply to this Work are set out in our licence referred to above.

Other than as permitted in any relevant BMJ Author's Self Archiving Policies, I confirm this Work has not been accepted for publication elsewhere, is not being considered for publication elsewhere and does not duplicate material already published. I confirm all authors consent to publication of this Work and authorise the granting of this licence.

For peer review only

Stress Among Medical Students: bifactor measurement model of the University Stress Scale

Igor Portoghese, PhD, Fabio Porru, MD, Maura Galletta, PhD, Marcello Campagna, MD, Alex Burdorf, PhD

I. Portoghese is Research Fellow, Department of Medical Science and Public Health, University of Cagliari, Monserrato, Italy; email: igor.portoghese@gmail.com; ORCID: http://orcid.org/0000-0003-3700-8594.

F. Porru is Research Assistant, Department of Public Health, Erasmus MC, Rotterdam, Netherlands; email: f.porru@erasmusmc.nl; ORCID: https://orcid.org/0000-0001-9202-6168

M. Galletta is Assistant Professor, Department of Medical Science and Public Health, University of Cagliari, Monserrato, Italy; email: maura.galletta@gmail.com ;ORCID: https://orcid.org/0000-0002-0124-4248.

M. Campagna is Associate Professor, Department of Medical Science and Public Health, University of Cagliari, Monserrato, Italy; email: Mam.campagna@gmail.com; ORCID: https://orcid.org/0000-0002-5277-8477

A. Burdorf is Full Professor, Department of Public Health, Erasmus MC, Rotterdam, Netherlands; email: a.burdorf@erasmusmc.nl; ORCID: https://orcid.org/0000-0003-3129-2862

Correspondence should be addressed to A. Burdorf. Email: a.burdorf@erasmusmc.nl; Department of Public Health, Erasmus MC, office Na2412, Dr.Molewaterplein 40, 3015 GD Rotterdam, The Netherlands.

Word count: 2792 words.

For peer review only

ABSTRACT

Objectives. The main purpose of the current study was to investigate the psychometric properties of the Italian version of the University Stress Scale (USS) among Italian medical students, by testing a bifactor measurement model adopting the Exploratory Structural Equation Modeling (ESEM) framework.

Design, setting and participants. A cross-sectional observational study based on data from an online cross-sectional survey filled by 1858 Italian medical students.

Outcome measures. University Stress, student stress and psychological distress were measured using respectively the USS, the Effort-Reward Imbalance Student Questionnaire (ERI-SQ), and the Kessler-10 (K10).

Results. Results showed that a bifactor-ESEM solution provided excellent levels of fit to the data. Our results suggest that the modified version of 19 items of the Italian version of the USS does not have a simple unidimensional structure. Specifically, the USS should be considered as composed by a global university stress factor and three specific factors, Academic, Equity, and Practical. Evidence of convergent validity was observed by analyzing the correlations between the USS and ERI-SQ (ranging from -.35 to .37, all $p < .01$). Finally, results showed that considering a clinical cut-off of 12.5 on the USS, 59.70% of medical students reported stress levels in the clinical range.

Conclusion. Finally, the Italian version of the USS is a valid, reliable and robust instrument for the measurement of stress among Italian medical students.

Keywords: academic stress, psychometric, bifactor modeling, bifactor exploratory structural equation modeling.

For peer review only

Strengths and limitations of this study

1. First study that examined a bifactor solution of the University Stress Scale
2. Robust instrument for the measurement of stress among Italian medical students
3. Lack of predictive validity
4. Lack of a test–retest reliability

For peer review only

INTRODUCTION

Mental health is a leading cause of disability and loss of health globally [1]. Mainly, mental health and well-being of young individuals has become a public health concern in many countries, [2]. In the last decade, many studies showed how the incidence of mental health problems among university students is directly associated with the increase of academic stress, [3]. High levels of stress and burnout have been described in higher education students worldwide, [4–8]. Recently, Deasy and colleagues emphasized that the student population is at higher risk of psychological distress when compared to the general population, [9].

Among University students, special attention was given to medical students, [2]. In the last decade, an increasing amount of research has dealt with investigating what makes students' lives more stressful. In fact, medical training has been recognized as a highly stressful experience for medical students, [10,11]. A recent review showed that stress levels among preclinical students varied between 20.9 and 90%, [12]. Several studies showed that the stress of medical training has an impact on the physical and mental health of medical students, [8,12–16]. Furthermore, many academic institutions are implementing interventions aimed at developing coping strategies in students exposed to chronic stressors of medical training, [17].

In this sense, identifying the major stressors within the academic context represents the first step in order to tackle efficiently psychological distress. In the last decade many studies investigated the sources of stress among medical students, founding that the most common ones are: (1) lack of practical skills, (2) feel pressured to get perfect grades, (3) fear of failing, (4) high workload (e.g. long hours of study), (5) low free time, (6) quality of the relationships with academic and clinical staff, (7) parental expectations, (8) frequency of examinations, [11,18–20].

For peer review only

Many authors adopted and extended original measures of stress, for example, by adapting work-related stress measures to the university context. Recently, Stallman and Hurst developed the University Stress Scale, [21]. According to the authors, this is a "screening measure that captures the cognitive appraisal of demands across the range of environmental stressors experienced by students". In addition, the authors proposed a (clinical-based) cut-off point as an indicator of the association of levels of stress and mental health problems risk, [21].

Stallman and Hurst defined the USS as composed of 6 factors: (1) academic, concerning coursework demands, procrastination and study/life balance; (2) equity, concerning discrimination, sexual orientation issues, and language/cultural issues; (3) parenting, concerning parenting issues and childcare; (4) relationships, concerning relationships with family, friends and partner, and relationship break-down; (5) practical, concerning finances and money problems, housing/accommodation, and transport; and (6) health, concerning mental and physical health problems, [21].

Concerning the multidimensional structure of the USS, Stallman and Hurst treated the USS as unidimensional in calculating cut-off criteria, [21]. In this sense, a bifactor measurement model should represent the better methodological strategy in analyzing the structural representation of this measure, [22–27]. In fact, in a bifactor measurement model items correlation can be accounted for by (1) a general factor (G-factor) representing the broad construct of the USS, and (2) a set of group factors representing the specific subdomains of the USS.

Furthermore, considering the highly restrictive confirmatory factor analysis (CFA) assumptions and the linked risk of overestimation of factor correlations, we adopted the Exploratory Structural Equation Modeling (ESEM) framework, [28]. It is a combination of Exploratory Factor Analysis and CFA, with less restrictive constraints. Specifically, adopting

For peer review only

the ESEM framework, items are allowed to load on all the factors, [29]. According to many authors, ESEM has been found to represent the factor structure of multidimensional constructs more effectively than CFA, [28,29]. ESEM tends to result in better fit and more accurate estimates, including more realistically estimated factor correlations, [28,29].

Therefore, the purpose of the current study was to a) investigate the psychometric properties of the Italian version of the USS among Italian medical students, and b) identify cut-offs of the USS to recognize students who are at risk of developing a mental illness.

METHODS

Participants and Procedure

Participants in this study were 1858 Italian medical students. The sample was recruited through a public announcement at electronic learning platforms for students and university students' associations. The web platforms contained an invitation to join an online survey entitled "UniCares - Health Promoting University". The first page of the online protocol described the study's objectives, the inclusion criteria, and the ethical issues underpinning the study. Participants were informed that their involvement in the study was voluntary and anonymous and that no information that could identify them would be collected. Only individuals who agreed to the study's conditions completed the survey. The online survey was implemented with LimeSurvey®. This study was developed in accordance with the ethical standards of the institutional and/or national research committee and with the 1964 Helsinki declaration and its later amendments.

Measures

University Stress Scale. The USS is a 21-item screening measure that captures the cognitive appraisal of demands across the range of environmental stressors experienced by

For peer review only

students, [21]. Students are asked to rate on a 4-point Likert scale ranging from “not at all” to “constantly”, the degree to which six stressors (academic, equity, parenting, relationships, practical, and health) were a source of stress in the previous month. The English version was first translated into Italian by a bilingual translator; then this version was translated back into English by another bilingual translator. Both versions were compared, and discrepancies were discussed and changed as needed. Specifically, two items from the original version concerning “parenting” were omitted from this study as in Italy the mean age of women at childbirth is 31.8 and 35.3 for men, whereas the mean age of Italian University students is 22, [30]. Thus, a version with 19 items was used for this study.

Students stress. The Effort-Reward Imbalance Student Questionnaire (ERI-SQ) was used for measuring university stress. The Italian version of the ERI-SQ is made up of 12 items that constitute three scales: Effort (EFF; 2 items; example: “I have constant time pressure due to a heavy study load”), Rewards (REW; 5 items; example: “I receive the respect I deserve from my supervisors/teachers”), and Over-commitment (OC; 6 items; example: “As soon as I get up in the morning I start thinking about study problems”), [31]. All items are scored on a 4-point rating scale ranging from 1 (strongly disagree) to 4 (strongly agree). Internal consistency was .69 for reward, and .78 for over-commitment.

Psychological Distress. The Kessler 10 (K10) is a brief measure of non-specific psychological distress that discriminates between cases and non-cases of depression and

For peer review only

anxiety disorders, [32,33]. Its clinical cut-off (K10≥ 20) in the student population makes it suitable to evaluate the clinical utility of the USS among university students [6]. Internal consistency was α = .90.

Patient and public involvement

No patient and public involved.

Statistical analyses

All models were estimated using the robust maximum likelihood (MLR) estimator in MPlus 7.3, [34]. In the first stage, CFA, Bifactor-CFA, ESEM, and Bifactor-ESEM were estimated. In first-order CFA, items were allowed to define their a priori factor, no cross-loading was allowed, and all factors were allowed to correlate. In Bifactor-CFA, all items were allowed to define one general factor (G-factor) and a priori specific factors (S-factors). No cross-loading was allowed, and all factors were specified as orthogonal. In first-order ESEM, oblique target rotation was used to allow the free estimation of the main loadings of the items on the a priori factors, while all cross-loadings were estimated but targeted to be as close to zero as possible. Finally, the Bifactor-ESEM solution was estimated using orthogonal target rotation, resulting in the free estimation of items loadings on the G-factor and on one out of S-factors, while all cross-loadings were freely estimated but targeted to be as close to zero as possible.

Following Morin and colleagues, we began comparing CFA and ESEM solutions [35,36]. In this comparison, as long as the factors remain well-defined by strong target factor loadings, the key issue is related to the factor correlations. Statistical evidence that ESEM tends to provide more exact estimates of true factor correlations suggests that ESEM should be retained whenever the results show a discrepant pattern of factor correlations [37]. Otherwise, the CFA model should be preferred based on parsimony. Then, the second comparison involves contrasting the retained model with its bifactor counterpart (bifactor-

For peer review only

CFA or bifactor-ESEM). Here, the key elements favouring a bifactor representation are the observation of a G-factor that is well-defined by strong factor loadings, and the observation of reduced cross-loadings in bifactor-ESEM compared to ESEM. A simplified representations of specified models is showed in figure 1.

For all models, we reported standardized factor loadings (λ: representing the strength of association between each specific item and the underlying factors) and model-based omega coefficients of composite reliability. We relied on the following common goodness-of-fit indices: the chi-square ($\chi2$), the comparative fit index (CFI), the Tucker-Lewis Index (TLI), the root mean square error of approximation (RMSEA). In interpreting these fit indices, we considered values > 0.90 and 0.95 for the CFI and TLI respectively, indicating adequate and excellent fit to the data, whereas values smaller than 0.08 or 0.06 for the RMSEA as acceptable and excellent model fit, [28,38,39].

Furthermore, we tested for convergent validity. Specifically, convergent validity is confirmed inspecting the correlation of USS with another conceptually and theoretically linked measure, the ERI student version. According to Cohen (1988), coefficients ≤ .29 were considered low, .3 to .49 moderate and correlations ≥ .5 as high.

Finally, following Stallman and Hurst, procedure, we determined the optimal cut-off point by analyzing the Receiver Operating Characteristic (ROC), [21]. Specifically, we compared the USS to the clinical cut-off recommended on the K10 for university students (K10≥ 20). We calculated sensitivities, specificities, positive (PPV) and negative predictive values (NPV) for the USS. We calculated the areas under the ROC curve (AUC) with 95% confidence intervals. For ROC analyses, we used R, [40].

RESULTS

For peer review only

Table 1 presents the goodness-of-fit indices and information criteria associated with each of the estimated models. The one factor-CFA, and the five factor-CFA demonstrated inadequate fit.

The ESEM solution did not converge after 10,000 iterations. In contrast, both the Bifactor-CFA and bifactor-ESEM provided excellent representation to the data according to all indices. Generally, comparisons of the models based on the information criteria supported the fit of the B-ESEM model over the corresponding B-CFA.

Table 1. Goodness of fit statistics of the Italian USS

Model	χ2	df	CFI	TLI	RMSEA (90% CI)	SRMR	AIC	BIC	ABIC
One-factor model	1458,38	68	0,56	0,49	,083(,079-,087)	0,068			
5 factor -CFA	711,77	94	0,80	0,74	,059(,055-,063)	0,051			
ESEM	*n.i.*	*n.i.*	*n.i.*	*n.i.*	*n.i.*	*n.i.*	*n.i.*	*n.i.*	*n.i.*
Bifactor-CFA	400,24	89	0,90	0,86	,043(,039-,047)	0,042			
Bifactor-ESEM	95,42	39	0,98	0,94	,028(,021-,035)	0,011			

Note: n = 1858; χ2 = Satorra-Bentler scaled chi-square, CFA = Confirmatory Factor Analysis; ESEM = Exploratory Structural Equation Modeling; df = Degrees of freedom; CFI = Comparative Fit Index; TLI = Tucker-Lewis Index; RMSEA = Root Mean Square Error of Approximation, 90% CI = 90% confidence interval for RMSEA; AIC = Akaike Information Criterial; BIC = Bayesian Information Criteria; ABIC = Sample-sized Adjusted BIC.

Bifactor ESEM solution provided excellent levels of fit to the data (CFI/TLI ≥0.900; RMSEA ≤0.060). The bifactor-ESEM solution (see table 2) reveals the presence of a G-factor that is relatively well defined by a majority of items (|λ| = 0.14 to 0.60, M = 0.32, ω = 0.77). Further examination of this solution reveals reasonably low cross-loadings, remaining lower than target loadings (|λ| = 0.01 to 0.16, M = 0.06).

For peer review only

Looking at the S-factors, the Academic (|λ| = 0.21 to 0.71, M = 0.51), Equity (|λ| = 0.36 to 0.50, M = 0.42) and Practical (|λ| = 0.30 to 0.60, M = 0.43) S-factors are relatively fair defined by most items, suggesting that these three S-factors retained specificity over and above that explained by the G-Factor. In contrast, the Relationships (|λ| = 0.01 to 0.96, M = 0.39) and Health (|λ| = 0.08 to 0.49, M = 0.29) S-factors appear to retain almost no specificity once the variance explained by the G-factor is taken into account, arguing against the added-value of this dimension.

Overall, these results support the superiority of the Bifactor-ESEM solution, which is retained for further analyses.

However, the model-based coefficients of composite reliability proved to be fair for the Academic (ω = 0.62), acceptable for the Relationships (ω = 0.69), and low for Equity (ω = 0.43), ad Practical (ω = 0.53) and Health S-Factors (ω = 0.51).

Table 2. Standardized parameter estimates from the bifactor-ESEM solutions

	G (λ)	S1 (λ)	S2 (λ)	S3 (λ)	S4 (λ)	S5 (λ)
USS1	0,15	0,60				
USS2	0,30	0,21				
USS17	0,38	0,72				
USS18	0,41		0,41			
USS19	0,19		0,50			
USS20	0,14		0,36			
USS11	0,60			-0,04		
USS12	0,46			0,18		
USS13	0,27			0,96		
USS14	0,21			0,46		
USS4	0,35				0,30	

For peer review

USS5	0,32				0,60	
USS6	0,20				0,40	
USS7	0,53					*0,31*
USS8	0,41					*0,49*
USS21	0,25					*0,08*

Note: λ = standardized factor loading; G = global factor; S1= specific factor: Academic; S2=Equity; S3=Relationships; S4=Practical; S5=Health;

Evidence of convergent validity was first obtained by analyzing the Pearson correlations between the USS and the ERI student version. As expected, positive and significant correlations were observed between USS and each of the ERI measures (ranging from -.35 to .37, all $p < .01$).

Finally, ROC curve analyses showed that a cut-off of 12.5 on the USS gave an area under the curve of .78 (95% CI .76-.81). The sensitivity and specificity of the test were 73.60% and 67.70%, respectively. The PPV of the test was 86.30%, and NPV of the test was 48.20% on psychological distress.

DISCUSSION

The main purpose of the present study was to investigate the psychometric properties of the Italian version of the USS. It was designed as a broad assessment of the categories of stressors that are experienced by university students. Stallman, and Hurst [21] suggested to use the USS as a general measure of stress, then considering it as a unidimensional measure. For this reason, our study aimed to examine the factor structure of the USS by using the ESEM approach, [35,36].

Our results confirmed that the USS does not have a simple unidimensional structure. When we tested different structures of the USS (CFA, ESEM, bifactor CFA and bifactor ESEM),

For peer review only

we found a clear indication that the USS reflects a combination of one global overarching university stress construct, coupled with five specific dimensions themselves characterized by cross-loadings. Consistent with a priori predictions, the bifactor-ESEM fitted the data better than traditional CFA. In this sense, our results show that part of the variance not explained by the G-factor (general university stress) is explained by specific relevant components. The variance left unexplained by this general university stress measure is associated with three S-factors: Academic, Equity, and Practical. These factors reflect specific sources of associated stress that are independent from the more global university stress. More specifically, the Academic subscale reflects items associated with academic/coursework demands, the equity subscale reflects items associated with discrimination, sexual orientation issues, and language/cultural issues. Finally, Practical subscale reflects items associated with housing/accommodation, finances and money problems, and transport. Conversely, items from the Relationship and Health subscales lost their specificity once G-factor was considered. Furthermore, support for convergent validity was found via the significant correlations with the ERI-SQ. The strength of their correlations suggests there is an overlap between these two measures of academic stress. Although the ERI-SQ has a different theoretical basis, both USS and ERI-SQ are claimed to measure the same general construct, [31,41].

The clinical cut-off score provides some indication of the level of stress that is likely to impact upon student performance, and possibly suggest mental health problems. Given that the majority of students (59.70%) reported stress levels in the clinical range, these findings also support previous research that highlighted the need for preventative interventions to enhance students' realistic appraisals and teach the coping mechanisms that are necessary to deal with stressful situations, [6]. This will help prevent the challenges that are associated

For peer review only

with university study from becoming distressing, thereby placing students at risk for the development of psychological disorders.

In general, outcomes from our study suggest that the Italian version of the USS is a multidimensional measure characterized by a global university stress factor, and by specific factors. Both G-factor and S-factors should be considered in measuring stress among university students when measured by the USS.

The present study has several limitations. First, we collected data from a convenience sample that reduced the generalizability of our results. However, considering the aim of the present study, this sample was considered appropriate. Second, this study lacks a test–retest reliability, which could test the consistency of the measures over time. Further research should explore these issues, along with measurement invariance across gender and different languages in order to increase our knowledge of university stress from a cross-cultural perspective.

AUTHOR CONTRIBUTIONS

IP, FB, MG, MC AB contributed to the conception and design of the study. IP, FB and MG and contributed to the development procedure of the Italian version of USS, including forward translation and back translation review. FP contributed to the acquisition of data. IP analyzed the data. FP wrote the first draft of the manuscript. IP and MG supervised the analysis. MG, AB and MC helped to draft and revise the manuscript. All authors read and approved the final manuscript.

COMPETING INTERESTS

The author(s) declared no potential conflicts of interest with respect to the research, authorship, and/or publication of this article.

For peer review only

FUNDING

The authors have not declared a specific grant for this research from any funding agency in the public, commercial or not-for-profit sectors.

AVAILABILITY OF DATA AND MATERIAL

Raw data pertaining to analyses performed in this study are available from the authors upon reasonable request.

For peer review only

REFERENCES

1. Wittchen HU, Jacobi F, Rehm J, Gustavsson A, Svensson M, Jönsson B, et al. The size and burden of mental disorders and other disorders of the brain in Europe 2010. Eur Neuropsychopharmacol J Eur Coll Neuropsychopharmacol. 2011 Sep;21(9):655–79.

2. Slavin SJ, Chibnall JT. Finding the Why, Changing the How: Improving the Mental Health of Medical Students, Residents, and Physicians. Acad Med J Assoc Am Med Coll. 2016;91(9):1194–6.

3. Blanco C, Okuda M, Wright C, Hasin DS, Grant BF, Liu S-M, et al. Mental health of college students and their non-college-attending peers: results from the National Epidemiologic Study on Alcohol and Related Conditions. Arch Gen Psychiatry. 2008 Dec;65(12):1429–37.

4. Verger P, Combes J-B, Kovess-Masfety V, Choquet M, Guagliardo V, Rouillon F, et al. Psychological distress in first year university students: socioeconomic and academic stressors, mastery and social support in young men and women. Soc Psychiatry Psychiatr Epidemiol. 2009 Aug;44(8):643–50.

5. Vázquez FL, Otero P, Díaz O. Psychological distress and related factors in female college students. J Am Coll Health J ACH. 2012;60(3):219–25.

6. Stallman HM. Psychological distress in university students: A comparison with general population data. Aust Psychol. 2010;45(4):249–57.

7. Portoghese I, Leiter MP, Maslach C, Galletta M, Porru F, D'Aloja E, et al. Measuring Burnout Among University Students: Factorial Validity, Invariance, and Latent Profiles

For peer review only

of the Italian Version of the Maslach Burnout Inventory Student Survey (MBI-SS). Front Psychol. 2018;9:2105.

8. Dyrbye LN, Thomas MR, Shanafelt TD. Systematic review of depression, anxiety, and other indicators of psychological distress among U.S. and Canadian medical students. Acad Med J Assoc Am Med Coll. 2006 Apr;81(4):354–73.

9. Deasy C, Coughlan B, Pironom J, Jourdan D, Mannix-McNamara P. Psychological distress and coping amongst higher education students: a mixed method enquiry. PloS One. 2014;9(12):e115193.

10. Toews JA, Lockyer JM, Dobson DJ, Brownell AK. Stress among residents, medical students, and graduate science (MSc/PhD) students. Acad Med J Assoc Am Med Coll. 1993 Oct;68(10 Suppl):S46-48.

11. Dyrbye LN, Thomas MR, Shanafelt TD. Medical student distress: causes, consequences, and proposed solutions. Mayo Clin Proc. 2005 Dec;80(12):1613–22.

12. Fares J, Al Tabosh H, Saadeddin Z, El Mouhayyar C, Aridi H. Stress, Burnout and Coping Strategies in Preclinical Medical Students. North Am J Med Sci. 2016 Feb;8(2):75–81.

13. Mazurkiewicz R, Korenstein D, Fallar R, Ripp J. The prevalence and correlations of medical student burnout in the pre-clinical years: a cross-sectional study. Psychol Health Med. 2012;17(2):188–95.

14. Matheson KM, Barrett T, Landine J, McLuckie A, Soh NL-W, Walter G. Experiences of Psychological Distress and Sources of Stress and Support During Medical Training: a

For peer review only

345 Survey of Medical Students. Acad Psychiatry J Am Assoc Dir Psychiatr Resid Train
346 Assoc Acad Psychiatry. 2016 Feb;40(1):63–8.

347 15. Dyrbye L, Shanafelt T. A narrative review on burnout experienced by medical students
348 and residents. Med Educ. 2016 Jan;50(1):132–49.

349 16. Bore M, Kelly B, Nair B. Potential predictors of psychological distress and well-being
350 in medical students: a cross-sectional pilot study. Adv Med Educ Pract. 2016;7:125–35.

351 17. Campo AE, Williams V, Williams RB, Segundo MA, Lydston D, Weiss SM. Effects of
352 LifeSkills training on medical students' performance in dealing with complex clinical
353 cases. Acad Psychiatry J Am Assoc Dir Psychiatr Resid Train Assoc Acad Psychiatry.
354 2008 Jun;32(3):188–93.

355 18. Slavin SJ, Schindler DL, Chibnall JT. Medical student mental health 3.0: improving
356 student wellness through curricular changes. Acad Med J Assoc Am Med Coll. 2014
357 Apr;89(4):573–7.

358 19. Reed DA, Shanafelt TD, Satele DW, Power DV, Eacker A, Harper W, et al.
359 Relationship of pass/fail grading and curriculum structure with well-being among
360 preclinical medical students: a multi-institutional study. Acad Med J Assoc Am Med
361 Coll. 2011 Nov;86(11):1367–73.

362 20. Bedewy D, Gabriel A. Examining perceptions of academic stress and its sources among
363 university students: The Perception of Academic Stress Scale. Health Psychol Open.
364 2015 Jul;2(2):2055102915596714.

365 21. Stallman HM, Hurst CP. The University Stress Scale: Measuring Domains and Extent of
366 Stress in University Students. Aust Psychol. 2016 Apr;51(2):128–34.

For peer review only

22. Rodriguez A, Reise SP, Haviland MG. Evaluating bifactor models: Calculating and interpreting statistical indices. Psychol Methods. 2016;21(2):137–50.

23. Reise SP, Morizot J, Hays RD. The role of the bifactor model in resolving dimensionality issues in health outcomes measures. Qual Life Res Int J Qual Life Asp Treat Care Rehabil. 2007;16 Suppl 1:19–31.

24. Reise SP. The rediscovery of bifactor measurement models. Multivar Behav Res. 2012;47(5):667–96.

25. Chen FF, West SG, Sousa KH. A Comparison of Bifactor and Second-Order Models of Quality of Life. Multivar Behav Res. 2006 Jun 1;41(2):189–225.

26. Chen FF, Hayes A, Carver CS, Laurenceau J-P, Zhang Z. Modeling general and specific variance in multifaceted constructs: a comparison of the bifactor model to other approaches. J Pers. 2012 Feb;80(1):219–51.

27. Canivez G. Bifactor modeling in construct validation of multifactored tests: Implications for understanding multidimensional constructs and test interpretation. In: Principles and methods of test construction: Standards and recent advancements [Internet]. In Schweizer K and DiStefano C (Eds.). Gottingen, Germany: Hogrefe Publishers; [cited 2019 Oct 18]. p. 247–271. Available from: https://www.scribd.com/document/386341939/Canivez-2016-Bifactor-Modeling-Chapter-12

28. Marsh HW, Morin AJS, Parker PD, Kaur G. Exploratory structural equation modeling: an integration of the best features of exploratory and confirmatory factor analysis. Annu Rev Clin Psychol. 2014;10:85–110.

For peer review only

29. Asparouhov T, Muthén B. Exploratory Structural Equation Modeling. Struct Equ Model Multidiscip J. 2009 Jul 14;16(3):397–438.

30. Istat. Condizioni di vita, reddito e carico fiscale delle famiglie [Internet]. [cited 2019 Jun 25] p. 25. Available from: https://www.istat.it/it/files//2018/12/Report-Reddito-Condizioni-di-vita-2017.pdf

31. Portoghese I, Porru F, Galletta M, Burdorf A, Campagna M. Stress among University Students: Factorial Structure and Measurement Invariance of the Italian Version of the Effort-Reward Imbalance Student Questionnaire. 2019 Press.

32. Furukawa TA, Kessler RC, Slade T, Andrews G. The performance of the K6 and K10 screening scales for psychological distress in the Australian National Survey of Mental Health and Well-Being. Psychol Med. 2003;33(2):357–62.

33. Kessler RC, Andrews G, Colpe LJ, Hiripi E, Mroczek DK, Normand SLT, et al. Short screening scales to monitor population prevalences and trends in non-specific psychological distress. Psychol Med. 2002;32(6):959–76.

34. Muthen L, Muthen B. Mplus Version 7 User's Guide: Version 7. Muthen & Muthen; 2012. 850 p.

35. Morin AJS, Arens AK, Tran A, Caci H. Exploring sources of construct-relevant multidimensionality in psychiatric measurement: A tutorial and illustration using the Composite Scale of Morningness. Int J Methods Psychiatr Res. 2016;25(4):277–88.

36. Morin AJS, Arens AK, Marsh HW. A Bifactor Exploratory Structural Equation Modeling Framework for the Identification of Distinct Sources of Construct-Relevant

For peer review only

Psychometric Multidimensionality. Struct Equ Model Multidiscip J. 2016 Jan 2;23(1):116–39.

37. Asparouhov T, Muthén B, Morin AJS. Bayesian structural equation modeling with cross-loadings and residual covariances: Comments on Stromeyer et al. J Manag. 2015;41(6):1561–77.

38. Marsh HW, Hau K-T, Grayson D. Goodness of Fit in Structural Equation Models. In: Contemporary psychometrics: A festschrift for Roderick P McDonald. Mahwah, NJ, US: Lawrence Erlbaum Associates Publishers; 2005. p. 275–340. (Multivariate applications book series).

39. Hu L, Bentler PM. Cutoff criteria for fit indexes in covariance structure analysis: Conventional criteria versus new alternatives. Struct Equ Model Multidiscip J. 1999 Jan 1;6(1):1–55.

40. R Foundation for Statistical Computing, Vienna, Austria. R Core Team. R: A language and environment for statistical computing [Internet]. [cited 2019 Oct 21]. Available from: http://www.R-project.org/

41. Wege N, Li J, Muth T, Angerer P, Siegrist J. Student ERI: Psychometric properties of a new brief measure of effort-reward imbalance among university students. J Psychosom Res. 2017;94:64–7.

For peer review only

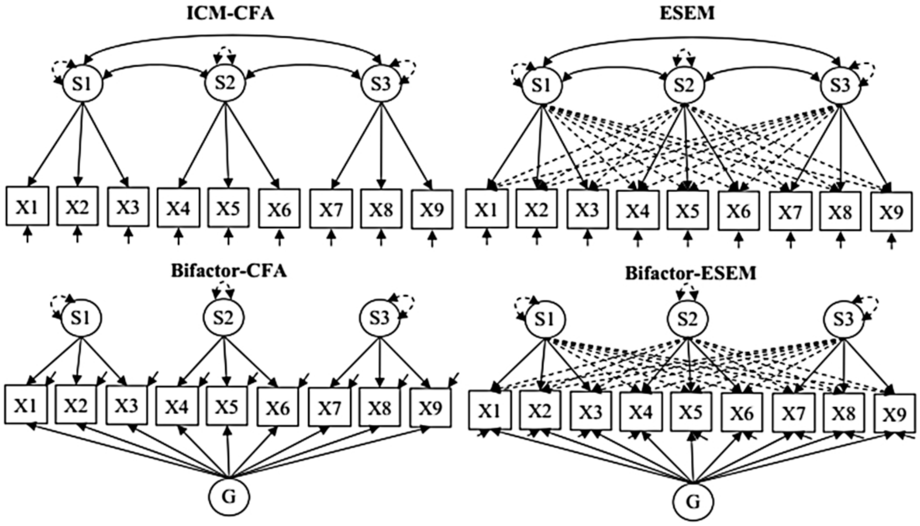

Figure 1. Simplified representations of specified models.

268x150mm (300 x 300 DPI)

BMJ Open

Stress Among Medical Students: Factor Structure of the University Stress Scale Among Italian Students

Journal:	*BMJ Open*
Manuscript ID	bmjopen-2019-035255.R1
Article Type:	Original research
Date Submitted by the Author:	03-Jun-2020
Complete List of Authors:	Portoghese, Igor; University of Cagliari, Medical Sciences and Public Health<br>Porru, Fabio; Erasmus Medical Center, Public Health; University of Cagliari, Medical Sciences and Public Health<br>Galletta, Maura; Universita degli Studi di Cagliari, Medical Sciences and Public Health<br>Campagna, Marcello; University of Cagliari, Medical Sciences and Public Health<br>Burdorf, Alex; Erasmus Medical Center, Public Health
**Primary Subject Heading**:	Public health
Secondary Subject Heading:	Medical education and training, Mental health
Keywords:	EDUCATION & TRAINING (see Medical Education & Training), PUBLIC HEALTH, PREVENTIVE MEDICINE

SCHOLARONE™
Manuscripts

I, the Submitting Author has the right to grant and does grant on behalf of all authors of the Work (as defined in the below author licence), an exclusive licence and/or a non-exclusive licence for contributions from authors who are: i) UK Crown employees; ii) where BMJ has agreed a CC-BY licence shall apply, and/or iii) in accordance with the terms applicable for US Federal Government officers or employees acting as part of their official duties; on a worldwide, perpetual, irrevocable, royalty-free basis to BMJ Publishing Group Ltd ("BMJ") its licensees and where the relevant Journal is co-owned by BMJ to the co-owners of the Journal, to publish the Work in this journal and any other BMJ products and to exploit all rights, as set out in our licence.

The Submitting Author accepts and understands that any supply made under these terms is made by BMJ to the Submitting Author unless you are acting as an employee on behalf of your employer or a postgraduate student of an affiliated institution which is paying any applicable article publishing charge ("APC") for Open Access articles. Where the Submitting Author wishes to make the Work available on an Open Access basis (and intends to pay the relevant APC), the terms of reuse of such Open Access shall be governed by a Creative Commons licence – details of these licences and which Creative Commons licence will apply to this Work are set out in our licence referred to above.

Other than as permitted in any relevant BMJ Author's Self Archiving Policies, I confirm this Work has not been accepted for publication elsewhere, is not being considered for publication elsewhere and does not duplicate material already published. I confirm all authors consent to publication of this Work and authorise the granting of this licence.

For peer review only

Stress Among Medical Students: Factor Structure of the University Stress Scale Among Italian Students

Igor Portoghese, PhD, Fabio Porru, MD, Maura Galletta, PhD, Marcello Campagna, MD, Alex Burdorf, PhD

I. Portoghese is Research Fellow, Department of Medical Science and Public Health, University of Cagliari, Monserrato, Italy; email: igor.portoghese@gmail.com; ORCID: http://orcid.org/0000-0003-3700-8594.

F. Porru is Scientific Researcher, Department of Public Health, Erasmus MC, Rotterdam, Netherlands; email: f.porru@erasmusmc.nl; ORCID: https://orcid.org/0000-0001-9202-6168

M. Galletta is Associate Professor, Department of Medical Science and Public Health, University of Cagliari, Monserrato, Italy; email: maura.galletta@gmail.com ;ORCID: https://orcid.org/0000-0002-0124-4248.

M. Campagna is Associate Professor, Department of Medical Science and Public Health, University of Cagliari, Monserrato, Italy; email: mam.campagna@gmail.com; ORCID: https://orcid.org/0000-0002-5277-8477

A. Burdorf is Full Professor, Department of Public Health, Erasmus MC, Rotterdam, Netherlands; email: a.burdorf@erasmusmc.nl; ORCID: https://orcid.org/0000-0003-3129-2862

Correspondence should be addressed to A. Burdorf. Email: a.burdorf@erasmusmc.nl; Department of Public Health, Erasmus MC, office Na2412, Dr.Molewaterplein 40, 3015 GD Rotterdam, The Netherlands.

Word count: 4150 words.

For peer review only

ABSTRACT

Objectives. The main purpose of the current study was to investigate the psychometric properties of the Italian version of the University Stress Scale (USS) among Italian medical students.

Design, setting and participants. A cross-sectional observational study based on data from an online cross-sectional survey from 11th to the 23rd of December 2018. A total of 1858 Italian medical students participated in the study.

Outcome measures. We measured perceived stress among medical students using the USS, the Effort-Reward Imbalance Student Questionnaire (ERI-SQ), and the Kessler-10 (K10).

Results. Results showed that a bifactor-ESEM solution provided excellent levels of fit to the data. Our results suggest that the modified version of 19 items of the Italian version of the USS does not have a simple unidimensional structure. Overall, an inspection of ancillary indices (omega indices, explained common variance, and percentage of uncontaminated correlations) revealed that these were too low to suggest the use of the USS as a composite measure of university stress. We tested an alternative unidimensional short form (eight items; USS-S) that assessed all the five sources of stress. This version provided good fit to the data. Evidence of convergent validity of the USS-S was observed by analyzing the correlations between the USS and ERI-SQ (ranging from -.34 to .37, all $p < .01$). Finally, based on the clinical cut-off recommended on the K10, results from Receiver Operating Characteristic showed that considering the clinical cut-off of the USS is 7.5 and that 59.70% of medical students reported stress levels in the clinical range.

For peer review only

Conclusion. Finally, our results showed a lack of support for using the USS to measure a general university stress factor, as the general USS factor accounted for little variance in our sample. In this sense, stress scores among Italian students can be better assessed by the use of the USS-S.

Keywords: academic stress, psychometric, bifactor modeling, bifactor exploratory structural equation modeling.

For peer review only

Strengths and limitations of this study

1. First study that examined a bifactor solution of the University Stress Scale
2. Short version for the measurement of general stress among Italian medical students
3. Lack of predictive validity
4. Lack of a test–retest reliability

For peer review only

INTRODUCTION

Mental health problems are a leading cause of disability and loss of health globally, [1]. Mainly, mental health problems and lack of well-being of young individuals have become a public health concern in many countries, [2]. In the last decade, many studies showed how the incidence of mental health problems among university students is directly associated with the increase of academic stress, [3-5]. High levels of stress and burnout have been described in higher education students worldwide, [6-10]. Recently, Deasy and colleagues emphasized that the student population is at higher risk of psychological distress when compared to the general population, [11]. Academic stress is an important variable that has received researchers' attention in the last 20 years. Rooted in the Lazarus and Folkman's [12] transactional model of stress, academic stress is conceived as a specific relationship between the student and the academic environment that is appraised by the student as taxing or exceeding his or her resources and endangering his or her well-being. Among the main sources of academic stress, there are attending lessons, study overload, respecting deadlines, assessments, financial demands, social pressures, and lack of balance between university and private life [13].

Among university students, special attention was given to medical students, [2]. In the last decade, an increasing amount of research has dealt with investigating what makes students' lives more stressful. In fact, medical training has been recognized as a highly stressful experience for medical students, [14,15]. A recent review showed that stress prevalence among preclinical students varied between 20.9% and 90%, [16]. Several studies showed that the stress of medical training has an impact on the physical and mental health of medical students, [10,15–19]. Furthermore, many academic institutions are implementing interventions aimed at developing coping strategies in students exposed to chronic stressors of medical training, [20].

For peer review only

In this sense, identifying the major stressors within the academic context represents the first step in order to tackle efficiently psychological distress. The most common sources of stress among medical students are: (1) lack of practical skills, (2) feel pressured to get perfect grades, (3) fear of failing, (4) high workload (e.g. long hours of study), (5) low free time, (6) quality of the relationships with academic and clinical staff, (7) parental expectations, (8) frequency of examinations, [14,21–23].

Many authors adopted and extended measures of stress, for example, by adapting work-related stress measures to the university context. Recently, Stallman and Hurst developed the University Stress Scale, [24]. According to the authors, this is a "screening measure that captures the cognitive appraisal of demands across the range of environmental stressors experienced by students". In addition, the authors proposed a (clinical-based) cut-off point as an indicator of the association of levels of stress and mental health problems risk, [24].

According to Stallman and Hurst [24], the USS was developed with three central purposes: (1) providing a broad measure of different categories of perceived stressors among university students instead of assessing symptoms of stress; (b) not restricting students' perception "of specific demands and their cognitive appraisal of situations within each category"; and (c) providing a reliable self-report measure of perceived stressors among university student population for quickly identifying academic stressors and planning interventions.

Stallman and Hurst defined the USS as composed of six factors: (1) academic, concerning coursework demands, procrastination and study/life balance; (2) equity, concerning discrimination, sexual orientation issues, and language/cultural issues; (3) parenting, concerning parenting issues and childcare; (4) relationships, concerning relationships with family, friends and partner, and relationship break-down; (5) practical,

For peer review only

concerning finances and money problems, housing/accommodation, and transport; and (6) health, concerning mental and physical health problems, [24].

Concerning the multidimensional structure of the USS, Stallman and Hurst treated the USS as unidimensional in calculating cut-off criteria, [24]. In its theorization, it is not clear if it should be considered as a single unidimensional construct or a collection of different factors. The authors found support for a six factors solution (17 items), but they suggested that "the strong reliability and validity of the complete measure warrants the inclusion of all items in this instrument", [24].

In this sense, a bifactor measurement model should represent the better methodological strategy in analyzing the structural representation of this measure, [25–30]. In fact, in a bifactor measurement model items correlation can be accounted for by (1) a general factor (G-factor) representing the broad construct of the USS, and (2) a set of group factors representing the specific subdomains of the USS.

Furthermore, considering the highly restrictive confirmatory factor analysis (CFA) assumptions and the linked risk of overestimation of factor correlations, we adopted the Exploratory Structural Equation Modeling (ESEM) framework, [31]. It is a combination of Exploratory Factor Analysis and CFA, with less restrictive constraints. Specifically, adopting the ESEM framework, items are allowed to load on all the factors, [31]. According to many authors, ESEM has been found to represent the factor structure of multidimensional constructs more effectively than CFA, [30,31]. ESEM tends to result in better fit and more accurate estimates, including more realistically estimated factor correlations, [30,31].

Therefore, the purpose of the current study was to a) investigate the psychometric properties of the Italian version of the USS among Italian medical students, and b) identify clinical cut-offs of the USS to recognize students who are at risk of developing a mental illness.

For peer review only

METHODS

Participants and Procedure

The target population was medical students in Italy. In Italy, there are 34 Courses of Medicine and Surgery for an estimated population of 154000 students), [32]. Admission to the degree course in Medicine and Surgery is dependent on the results of a national entrance exam. The Italian legislation lays down six years of study for the degree course in Medicine and Surgery including four years for compulsory Internship/vocational training starting in the third year.

A total of 1858 Italian medical students participated in this cross-sectional study. The sample was recruited through a public announcement at electronic learning platforms for students and university students' associations. The web platforms contained an invitation to join an online survey entitled "UniCares - Health Promoting University". The first page of the online protocol described the study's objectives, the time necessary to complete the survey (less than 10 min), the inclusion criteria (being a university student of course in Medicine and Surgery in Italy), and the ethical issues underpinning the study. Participants were informed that their involvement in the study was voluntary and anonymous and that no information that could identify them would be collected. Only individuals who agreed to the study's conditions completed the survey. Furthermore, to ensure anonymity, we did not register the IP address, neither requested any another sensitive data. Finally, the research team did not offer any incentives to increase recruitment neither played any active role in selecting and/or targeting specific subpopulations of students.

For peer review only

The online survey was implemented with LimeSurvey® and was available online from 11th to the 23rd of December, 2018. The questionnaire investigated sociodemographic characteristics of the respondents, and three measures of perceived stress.

This study was developed in accordance with the ethical standards of the institutional and/or national research committee and with the 1964 Helsinki declaration and its later amendments.

Measures

University Stress Scale. The USS is a 21-item screening measure that captures the cognitive appraisal of demands across the range of environmental stressors experienced by students, [24]. Students are asked to rate on a 4-point Likert scale ranging from “not at all” to “constantly”, the degree to which six stressors (academic, equity, parenting, relationships, practical, and health) were a source of stress in the previous month. The English version was first translated into Italian by a bilingual translator, and then this version was translated back into English by another bilingual translator. The two versions were compared, and discrepancies were discussed and changed as needed. Specifically, we removed the parenting subscale (two items) as in Italy the mean age of women at childbirth is 31.8 and 35.3 for men, whereas the mean age of Italian University students is 22, [33]. Thus, a version with 19 items was used for this study.

Students stress. The Effort-Reward Imbalance Student Questionnaire (ERI-SQ) was used for measuring university stress. The Italian version of the ERI-SQ is made up of 12 items that constitute three scales: Effort (EFF; 2 items; example: “I have constant time pressure due to a heavy study load”), Rewards (REW; 5 items;

For peer review only

example: "I receive the respect I deserve from my supervisors/teachers"), and Over-commitment (OC; 6 items; example: "As soon as I get up in the morning I start thinking about study problems"), [34]. All items are scored on a 4-point rating scale ranging from 1 (strongly disagree) to 4 (strongly agree). The internal consistency for reward and for over-commitment in this sample were adequate, with a McDonald's omega value respectively of ω = .69, and ω = .79

Psychological Distress. The Kessler 10 (K10) is a brief measure of non-specific psychological distress that discriminates between cases and non-cases of depression and anxiety disorders, [35,36]. Its clinical cut-off (K10≥ 20) in the student population makes it suitable to evaluate the clinical utility of the USS among university students [8]. McDonald's omega was ω = .92.

Patient and public involvement

No patient and public involved.

Statistical analyses

Descriptive statistics were used for sociodemographic information of the population. All models were estimated using the weighted least squares mean and variance adjusted (WLSMV) estimator in MPlus 7.3, [37,38]. In the first stage, unidimensional, first and second-order confirmatory factor analysis (CFA), Bifactor-CFA, ESEM, and Bifactor-ESEM were estimated. In first-order CFA, items were allowed to define their a priori factor, no cross-loading was allowed, and all factors were allowed to correlate. In second-order CFA,

For peer review only

we tested whether the five first-order factors converge into a second-order factor of university stress. In Bifactor-CFA, all items were allowed to define one general factor (G-factor) and a priori specific factors (S-factors). No cross-loading was allowed, and all factors were specified as orthogonal. In first-order ESEM, oblique target rotation was used to allow the free estimation of the main loadings of the items on the a priori factors, while all cross-loadings were estimated but targeted to be as close to zero as possible. Finally, the Bifactor-ESEM solution was estimated using orthogonal target rotation, resulting in the free estimation of items loadings on the G-factor and on one out of S-factors, while all cross-loadings were freely estimated but targeted to be as close to zero as possible.

Following Morin and colleagues, we began comparing CFA and ESEM solutions [39,40]. In this comparison, as long as the factors remain well-defined by strong target factor loadings, the key issue is related to the factor correlations. Statistical evidence that ESEM tends to provide more exact estimates of true factor correlations suggests that ESEM should be retained whenever the results show a discrepant pattern of factor correlations [41]. Otherwise, the CFA model should be preferred based on parsimony. Then, the second comparison involves contrasting the retained model with its bifactor counterpart (bifactor-CFA or bifactor-ESEM). Here, the key elements favoring a bifactor representation are the observation of a G-factor that is well-defined by strong factor loadings, and the observation of reduced cross-loadings in bifactor-ESEM compared to ESEM. A simplified representation of specified models is showed in figure 1.

For all models, we reported standardized factor loadings (λ: representing the strength of association between each specific item and the underlying factors) and model-based omega coefficients of composite reliability. We relied on the following common goodness-of-fit indices: the chi-square ($\chi2$), the comparative fit index (CFI), the Tucker-Lewis Index (TLI), the root mean square error of approximation (RMSEA). In interpreting these fit indices, we

For peer review only - http://bmjopen.bmj.com/site/about/guidelines.xhtml

considered values > 0.90 and 0.95 for the CFI and TLI respectively, indicating adequate and excellent fit to the data, whereas values smaller than 0.08 or 0.06 for the RMSEA as acceptable and excellent model fit, and the weighted root mean square residual (WRMR; ≤ 1.00 for acceptable), [31,42,42].

For model-based reliability, we assessed omega coefficients, including omega for the total score (ω), omega subscale (ω_S), omega hierarchical (ω_H), and omega hierarchical subscale (ω_{HS}), [25,26]. ω and ω_S provided information about the reliability of the total scale and subscales, respectively. ω_H provided information about the extent to which composite scale scores were interpretable as a measure of a single common factor. ω_{HS} indicated the viability of subscales after controlling the variance due to the general factor. Higher ω and ω_S scores represent better reliability of the total scale and subscales. Specifically, a ω_H value higher than .80 suggests that the majority of the reliable variance can be attributed to a single general factor, [25]. A lower ω_{HS} value suggests that a higher proportion of the USS score variance can be attributed to the general factor, [25]. Similarly, in order to determine dimensionality, we calculated the explained common variance for the general factor (ECV), the individual item explained common variance (IECV), and the per cent of uncontaminated correlations (PUC), [26,27]. The ECV is the percentage of common variance attributable to the general factor, [25]. An ECV ≥.70 indicates a strong general factor and suggests the common variance is unidimensional, [25]. The IECV is the percentage of item common variance attributable to a general factor, [43]. The PUC helps in determining whether the bifactor data are unidimensional or multidimensional, [24]. PUC > .70 indicates less bias in structural coefficients and thus indicating that the measure can be treated as unidimensional, [25]. According to Rodriguez and colleagues, when ECV is > .70 and PUC is > .70, “relative bias will be slight, and the common variance can be regarded as essentially unidimensional", (p. 232), [25].

For peer review only

As universally adopted cut-off points for the above indices are not available, we followed Reise and colleagues' suggestion to consider ECV>0.6, PUC > 0.7, and ω_H > 0.7 in accepting the USS "unidimensional enough" to warrant the use of a total score [27]. Furthermore, we tested for convergent validity. Specifically, convergent validity is confirmed inspecting the correlation of USS with another conceptually and theoretically linked measure, the ERI student version. According to Cohen, coefficients ≤ .29 were considered low, .3 to .49 moderate and correlations ≥ .5 as high, [44].

Finally, following Stallman and Hurst, procedure, we determined the optimal cut-off point by analyzing the Receiver Operating Characteristic (ROC), [24]. Specifically, we compared the USS to the clinical cut-off recommended on the K10 for university students (K10≥ 20). We calculated sensitivities, specificities, positive (PPV) and negative predictive values (NPV) for the USS. We calculated the areas under the ROC curve (AUC) with 95% confidence intervals. For ROC analyses, we used R, [45].

RESULTS

Sociodemographic

The sample for this research consisted of 71.5% females (n = 1329). Participants in this study ranged from 19 to 30 years of age (M = 23.03, SD = 2.17). 16.8% (313) were enrolled in preclinical courses (first and second year) and 83.2% (1545) in clinical courses (years 3, 4,5, and 6).

Psychometric properties of the USS

Table 1 presents the goodness-of-fit indices and information criteria associated with each of the estimated models. The one factor-CFA, the five factor-CFA, and the second-order CFA demonstrated inadequate fit. In contrast, the ESEM, the bifactor-CFA and bifactor-ESEM provided excellent representation to the data. However, according to all indices. Generally,

For peer review only

comparisons of the models based on the information criteria supported the fit of the B-ESEM model over the corresponding B-CFA.

Table 1. Goodness of fit statistics of the Italian USS

Model	χ2	df	CFI	TLI	RMSEA (90% CI)	WRMR
One-factor CFA	2671.97	152	0.65	0.61	0.094(0.091-0.097)	3.27
5-factor CFA	1570.48	142	0.84	0.77	0.073(0.070-0.076)	2.46
Second-order CFA	817.32	147	0.90	0.88	0.050(0.046-0.053)	1.84
One factor ESEM	2671.97	152	0.65	0.61	0.094(0.091-0.097)	3.27
5 factor ESEM	366.39	86	0.96	0.92	0.042(0.037-0.046)	0.96
Bifactor-CFA	750.72	134	0.92	0.89	0.049(0.046-0.053)	1.67
Bifactor-ESEM	192.84	72	0.98	0.96	0.30(0.25-0.035)	0.67

Note: n = 1858; χ2 = Satorra-Bentler scaled chi-square, CFA = Confirmatory Factor Analysis; ESEM = Exploratory Structural Equation Modeling; df = Degrees of freedom; CFI = Comparative Fit Index; TLI = Tucker-Lewis Index; RMSEA = Root Mean Square Error of Approximation, 90% CI = 90% confidence interval for RMSEA;WRMR = Weighted Least Squares Mean and Variance..

Bifactor ESEM solution provided excellent levels of fit to the data (CFI/TLI ≥0.90; RMSEA ≤0.060). The bifactor-ESEM solution (see table 2) reveals the presence of a G-factor that is

relatively well defined by a majority of items ($|\lambda|$ = 0.17 to 0.67, M = 0.38). Further examination of this solution reveals reasonably low cross-loadings, remaining lower than target loadings ($|\lambda|$ = 0.01 to 0.16, M = 0.06).

Looking at the S-factors, the Academic ($|\lambda|$ = 0.23 to 0.76, M = 0.55), Equity ($|\lambda|$ = 0.21 to 0.69, M = 0.48) and Practical ($|\lambda|$ = 0.33 to 0.68, M = 0.49) S-factors are relatively fair defined by most items, suggesting that these three S-factors retained specificity over and above that explained by the G-Factor. In contrast, the Relationships ($|\lambda|$ = 0.01 to 0.96, M = 0.43) and Health ($|\lambda|$ = 0.06 to 0.26, M = 0.16) S-factors appear to retain almost no specificity once the variance explained by the G-factor is taken into account, arguing against the added-value of this dimension. Overall, these results support the superiority of the Bifactor-ESEM solution, which is retained for further analyses.

For model-based reliability, we assessed omega coefficients. In this study, the ω for the general factor was 0.85, above the recommended threshold of .75, indicating that a general factor explained more than 85% of the reliable variance. On the contrary, the ω_S values were acceptable only for Relationships = 0.76 and Equity = 0.76, fair for Academic = 0.69, and low for Practical= 0.61, and Health S-Factors = 0.46. The ω_H value was 0.65, suggesting that 65% of the total USS variance could be attributed to the general factor. Concerning the ω_{HS} for the five specific factors were: academic=.53, relationships=.44, equity=.38, practical=.43, and health=.02.

Concerning the results about the ECV, IECV, and PUC, the common variance associated with the general factor was .38, below the .70 threshold The ECV for each specific factor for academic, relationships, equity practical, and health were .16, .15, .19, .11, and .01 respectively. The average IECV for the general USS factor was 0.46 and ranged from 0.07 to 0.99. Only 4 items (USS2, USS11, USS15, and USS16) showed IECV greater than 0.80. Thus, it suggested that, on average, items measured the general factor to a slightly weaker

For peer review only

degree than they measured the intended specific factor. Finally, the PUC was 0.82, greater than the suggested 0.70 threshold. However, while higher PUC has been shown decreased the importance of considering the ECV in determining measurement parameter bias, ECV in the present study is not high enough [25]. Overall, these three findings further provided us more information concerning the dimensionality of the USS suggesting that modelling the USS as a unidimensional instrument would lead to substantial measurement parameter bias, and then disqualifying the interpretation of the USS as primarily unidimensional. Therefore, we sought to develop an alternative unidimensional short form of the Italian version of the USS that would consider full coverage of the five sources of stress. In accomplishing this goal, we followed the procedure suggested by Stucky and colleagues [43]: (a) selecting IECV > 0.80, and (b) and items with the larger factor loading on the general factor. The items comprising the short form of the USS (USS-S) were: USS2, USS4, USS7, USS11, USS12, USS16, USS17, and USS18. We next assessed the unidimensionality of the USS-S derived from the bifactor ESEM and I-ECV analysis. The 8-items unidimensional CFA provided a good fit to the data: CFI= 0.94, TLI= 0.91; RMSEA=0.068 (95% CI 0.059-0.077), WRMR=1.64. Factor loadings ranged from 0.35 to 0.66, M = 0.52) and McDonald's ω was acceptable (0.69).

Table 2. Standardized parameter estimates from the bifactor-ESEM solutions of the USS and factor loadings from CFA solution of the USS-S

	G (λ)	S1 (λ)	S2 (λ)	S3 (λ)	S4 (λ)	S5 (λ)	USS-S
USS1	0.23	0.80	*0.00*	*-0.04*	*0.01*	*0.00*	
USS2	0.42	0.13	*-0.05*	*0.01*	*-0.12*	*0.00*	0.46
USS3	0.36	0.46	*0.06*	*0.06*	*0.11*	*0.07*	
USS15	0.32	0.02	*0.12*	*0.11*	*0.24*	*-0.01*	
USS16	0.80	0.03	*-0.04*	*-0.04*	*-0.12*	*-0.23*	0.66
USS17	0.54	0.56	*-0.06*	*-0.01*	*-0.02*	*0.02*	0.57
USS18	0.52	*0.10*	0.49	*-0.05*	*0.08*	*0.03*	0.54
USS19	0.27	*-0.01*	0.72	*0.05*	*-0.15*	*-0.04*	
USS20	0.18	*-0.10*	0.58	*-0.01*	*0.13*	*0.06*	

USS11	0.55	*-0.17*	*-0.10*	0.03	*0.13*	*0.18*	0.55
USS12	0.39	*0.02*	*0.05*	0.25	*0.08*	*0.14*	0.44
USS13	0.25	*0.01*	*-0.04*	0.95	*-0.05*	*-0.08*	
USS14	0.21	*-0.01*	*0.05*	0.61	*0.00*	*0.06*	
USS4	0.36	*0.00*	*0.01*	*-0.03*	0.39	*0.05*	0.35
USS5	0.32	*0.01*	*0.06*	*0.02*	0.64	*-0.07*	
USS6	0.18	*0.05*	*-0.03*	*-0.02*	0.49	*-0.04*	
USS7	0.52	*0.09*	*-0.03*	*0.05*	*-0.13*	0.52	0.57
USS8	0.38	*0.01*	*-0.02*	*-0.01*	*0.07*	0.47	
USS21	0.30	*-0.02*	*0.22*	*-0.06*	*-0.10*	0.23	

Note: λ = standardized factor loading; G = global factor; S1= specific factor: Academic; S2=Equity; S3=Relationships; S4=Practical; S5=Health; USS-S= University Stress Scale Short

Evidence of convergent validity was first obtained by analyzing the Pearson correlations between the USS-S and the ERI student version. As expected, significant correlations were observed between the USS-S and each of the ERI measures. Specifically, (ranging from -.34 to .37, all $p < .001$).

Finally, a ROC curve analysis using K10 >=20 as a criterion for possible mental health problems showed that a cut-off of 7.5 on the USS gave an area under the curve of .78 (95% CI .76-.80). The sensitivity and specificity of the test were 73.60% (95% CI 71.3% -75.9%) and 65.20% (95% CI 61.0 -69.40%), respectively. The PPV of the test was 85.60% (95% CI 83.60% - 87.60%), and NPV of the test was 49.40% (95% CI 45.60% -53.20%) on psychological distress.

DISCUSSION

The main purpose of the present study was to investigate the psychometric properties of the Italian version of the USS. It was designed as a broad assessment of the categories of

For peer review only

stressors that are experienced by university students. Stallman, and Hurst [24] suggested to use the USS as a general measure of stress, then considering it as a unidimensional measure. In order to gain a better understanding of the strengths of the USS as unidimensional scale, we tested several latent measurement models, including standard CFA, ESEM, bifactor ESEM, and bifactor CFA, [38,40].

Initially, our results showed that the bifactor ESEM provided the better fit to the data compared to the other tested models.

Specifically, we found that the USS is composed of a general stress factor and five specific factors, namely Academic, Relationships, Equity, Practical, and Health. However, when we considered the ancillary bifactor indices of dimensionality and model-based reliability, results provided us with further information that considering the Italian version of the USS as unidimensional may produce a biased measure of stress. The examination of omega indices, ECV and PUC suggests that the general factor did not account for enough variance to be considered unidimensional. Specifically, an ECV of .38 implies that most of the common variance (62%) is explained by the specific factors. In this sense, Stallman and Hurst [24] never considered this solution and our results suggested that it should be not ignored.

.

From a theoretical perspective, our findings do not support the existence of a measurable "overall university stress" construct as suggested by Stallman and Hurst, [24], and the USS is not a reliable measure of general university stress among Italian medical students. In this sense, considering IECV coefficients based on the bifactor ESEM solution, we derived and tested a short version of this measure (USS-S). This new version showed a clear unidimensional factor structure. This supports the use of the USS-S total score as a measure of the overall stress among medical students.

For peer review only

The items that comprise the shorter version concern study/life balance, procrastination, parental expectations, work, friendships, family relationships, finances and money problems, and mental health problems. All items cover the main sources of stress as theorized by Stallman and Hurst, [24].

Furthermore, support for convergent validity of the USS-S was found via the significant correlations with each domain of the ERI-SQ. Although the ERI-SQ has a different theoretical basis, both USS and ERI-SQ are claimed to measure the same general construct, [34,46]. Specifically, the USS-S was moderately positively correlated to the Effort subscale. According to the ERI theory, this measure was developed to capture general demands and obligations. The strength of their correlations suggests there is an overlap between these two measures of academic stress. However, further investigation is necessary for understanding the convergent validity of the USS-S.

The clinical cut-off score provides some indication of the level of stress that is likely to impact upon student performance, and possibly suggest mental health problems. Given that the majority of students (59.70%) reported stress levels in the clinical range, these findings also support previous research that highlighted the need for preventative interventions to enhance students' realistic appraisals and teach the coping mechanisms that are necessary to deal with stressful situations, [8]. This will help prevent the challenges that are associated with university study from becoming distressing, thereby placing students at risk for the development of psychological disorders.

In general, outcomes from our study suggest that the Italian version of the USS is a multidimensional measure characterized by a global university stress factor, and by specific factors. Both G-factor and S-factors should be considered in measuring stress among university students when measured by the USS. However, researchers interested in adopting this measure are strongly encouraged to verify its dimensionality during preliminary analyses.

For peer review only

The present study has several limitations. First, we collected data from a convenience sample that reduced the generalizability of our results. That implies that the principles of probability sampling were not followed and our results could be biased. However, considering the aim of the present study to assess the psychometric properties of the USS among Italian medical students, this sample was considered appropriate. Second, as we derived a shorter version of the USS, we strongly encourage future research to investigate psychometric properties of this version among other cultural groups. Third, this study lacks a test–retest reliability, which could test the consistency of the measures over time. Fourth, we did not investigate measurement invariance across gender and different languages. Future research should explore that in order to increase knowledge of university stress from a cross-cultural perspective. Finally, we did not investigate the predictive validity of the Italian version of the USS-S. Future research should explore, for example, the ability of the USS to predict psychological well-being among students.

AUTHOR CONTRIBUTIONS

IP, FB, MG, MC AB contributed to the conception and design of the study. IP, FB and MG and contributed to the development procedure of the Italian version of USS, including forward translation and back translation review. FP contributed to the acquisition of data. IP analyzed the data. FP wrote the first draft of the manuscript. IP and MG supervised the analysis. MG, AB and MC helped to draft and revise the manuscript. All authors read and approved the final manuscript.

COMPETING INTERESTS

The author(s) declared no potential conflicts of interest with respect to the research, authorship, and/or publication of this article.

For peer review only

FUNDING

The authors have not declared a specific grant for this research from any funding agency in the public, commercial or not-for-profit sectors.

AVAILABILITY OF DATA AND MATERIAL

Raw data pertaining to analyses performed in this study are available from the authors upon reasonable request.

Figure 1. Simplified representations of specified models.

REFERENCES

1. Wittchen HU, Jacobi F, Rehm J, Gustavsson A, Svensson M, Jönsson B, et al. The size and burden of mental disorders and other disorders of the brain in Europe 2010. Eur Neuropsychopharmacol J Eur Coll Neuropsychopharmacol. 2011 Sep;21(9):655–79.

2. Slavin SJ, Chibnall JT. Finding the Why, Changing the How: Improving the Mental Health of Medical Students, Residents, and Physicians. Acad Med J Assoc Am Med Coll. 2016;91(9):1194–6.

3. Blanco C, Okuda M, Wright C, Hasin DS, Grant BF, Liu S-M, et al. Mental health of college students and their non-college-attending peers: results from the National Epidemiologic Study on Alcohol and Related Conditions. Arch Gen Psychiatry. 2008 Dec;65(12):1429–37.

4. Pozos-Radillo BE, Preciado-Serrano M de L, Acosta-Fernández M, Aguilera-Velasco M de los Á, Delgado-García DD. Academic stress as a predictor of chronic stress in university students. Psicol Educ. 2014 Jun;20(1):47–52.

For peer review only

5. Pluut H, Curseu PL, Ilies R. Social and study related stressors and resources among university entrants: Effects on well-being and academic performance. Learn Individ Differ. 2015;37:262–268.

6. Verger P, Combes J-B, Kovess-Masfety V, Choquet M, Guagliardo V, Rouillon F, et al. Psychological distress in first year university students: socioeconomic and academic stressors, mastery and social support in young men and women. Soc Psychiatry Psychiatr Epidemiol. 2009 Aug;44(8):643–50.

7. Vázquez FL, Otero P, Díaz O. Psychological distress and related factors in female college students. J Am Coll Health J ACH. 2012;60(3):219–25.

8. Stallman HM. Psychological distress in university students: A comparison with general population data. Aust Psychol. 2010;45(4):249–57.

9. Portoghese I, Leiter MP, Maslach C, Galletta M, Porru F, D'Aloja E, et al. Measuring Burnout Among University Students: Factorial Validity, Invariance, and Latent Profiles of the Italian Version of the Maslach Burnout Inventory Student Survey (MBI-SS). Front Psychol. 2018;9:2105.

10. Dyrbye LN, Shanafelt T. A narrative review on burnout experienced by medical students and residents. Med Educ. 2016;50(1):132-149.

.

11. Deasy C, Coughlan B, Pironom J, Jourdan D, Mannix-McNamara P. Psychological distress and coping amongst higher education students: a mixed method enquiry. PloS One. 2014;9(12):e115193.

12. Lazarus RS, Folkman S. Stress, Appraisal, and Coping. 1984, New York: Springer.

For peer review only

13. Pitt, A., Oprescu, F., Tapia, G., & Gray, M. An exploratory study of students' weekly stress levels and sources of stress during the semester. Act Learn High Educ. 2018;19(1):61-75.

14 Hope V, Henderson M. Medical student depression, anxiety and distress outside North America: a systematic review. Med Edu. 2014; 48(10):963-979. .

15 Shiralkar MT, Harris TB, Eddins-Folensbee FF, Coverdal JH. A systematic review of stress-management programs for medical students. Acad Psychiatry. 2013;37(3):158-164..

16. Fares J, Al Tabosh H, Saadeddin Z, El Mouhayyar C, Aridi H. Stress, Burnout and Coping Strategies in Preclinical Medical Students. North Am J Med Sci. 2016 Feb;8(2):75–81.

17. Mazurkiewicz R, Korenstein D, Fallar R, Ripp J. The prevalence and correlations of medical student burnout in the pre-clinical years: a cross-sectional study. Psychol Health Med. 2012;17(2):188–95.

18. Matheson KM, Barrett T, Landine J, McLuckie A, Soh NL-W, Walter G. Experiences of Psychological Distress and Sources of Stress and Support During Medical Training: a Survey of Medical Students. Acad Psychiatry J Am Assoc Dir Psychiatr Resid Train Assoc Acad Psychiatry. 2016 Feb;40(1):63–8.

19. Bore M, Kelly B, Nair B. Potential predictors of psychological distress and well-being in medical students: a cross-sectional pilot study. Adv Med Educ Pract. 2016;7:125–35.

For peer review only

20. Campo AE, Williams V, Williams RB, Segundo MA, Lydston D, Weiss SM. Effects of LifeSkills training on medical students' performance in dealing with complex clinical cases. Acad Psychiatry J Am Assoc Dir Psychiatr Resid Train Assoc Acad Psychiatry. 2008 Jun;32(3):188–93.

21 Slavin SJ, Schindler DL, Chibnall JT. Medical student mental health 3.0: improving student wellness through curricular changes. Acad Med J Assoc Am Med Coll. 2014 Apr;89(4):573–7.

22 Reed DA, Shanafelt TD, Satele DW, Power DV, Eacker A, Harper W, et al. Relationship of pass/fail grading and curriculum structure with well-being among preclinical medical students: a multi-institutional study. Acad Med J Assoc Am Med Coll. 2011 Nov;86(11):1367–73.

23 Bedewy D, Gabriel A. Examining perceptions of academic stress and its sources among university students: The Perception of Academic Stress Scale. Health Psychol Open. 2015 Jul;2(2):2055102915596714.

24 Stallman HM, Hurst CP. The University Stress Scale: Measuring Domains and Extent of Stress in University Students. Aust Psychol. 2016 Apr;51(2):128–34.

25. Rodriguez A, Reise SP, Haviland MG. Evaluating bifactor models: calculating and interpreting statistical indices. Psychol Methods. 2016b;21(2):137-150. 26. Reise SP. The rediscovery of bifactor measurement models. Multivar Behav Res. 2012;47(5):667–96.

27. Chen FF, West SG, Sousa KH. A Comparison of Bifactor and Second-Order Models of Quality of Life. Multivar Behav Res. 2006 Jun 1;41(2):189–225.

For peer review only

28. Chen FF, Hayes A, Carver CS, Laurenceau J-P, Zhang Z. Modeling general and specific variance in multifaceted constructs: a comparison of the bifactor model to other approaches. J Pers. 2012 Feb;80(1):219–51.

29. Canivez G. Bifactor modeling in construct validation of multifactored tests: Implications for understanding multidimensional constructs and test interpretation. In: Principles and methods of test construction: Standards and recent advancements [Internet]. In Schweizer K and DiStefano C (Eds.). Gottingen, Germany: Hogrefe Publishers; [cited 2019 Oct 18]. p. 247–271. Available from: https://www.scribd.com/document/386341939/Canivez-2016-Bifactor-Modeling-Chapter-12

30. Marsh HW, Morin AJS, Parker PD, Kaur G. Exploratory structural equation modeling: an integration of the best features of exploratory and confirmatory factor analysis. Annu Rev Clin Psychol. 2014;10:85–110.

31. Asparouhov T, Muthén B. Exploratory Structural Equation Modeling. Struct Equ Model Multidiscip J. 2009 Jul 14;16(3):397–438.

32. Ministero dell’Istruzione dell’Università e della Ricerca. Portale dei dati dell’istruzione superiore [Internet]. [cited 2020 May 27]. Available from: http://dati.ustat.miur.it/dataset/iscritti

33. Istat. Condizioni di vita, reddito e carico fiscale delle famiglie [Internet]. [cited 2019 Jun 25] p. 25. Available from: https://www.istat.it/it/files//2018/12/Report-Reddito-Condizioni-di-vita-2017.pdf

34. Portoghese I, Porru F, Galletta M, Burdorf A, Campagna M. Stress among University Students: Factorial Structure and Measurement Invariance of the Italian

For peer review only

Version of the Effort-Reward Imbalance Student Questionnaire. BMC Psychol. 2019;7(1), 68.

35. Furukawa TA, Kessler RC, Slade T, Andrews G. The performance of the K6 and K10 screening scales for psychological distress in the Australian National Survey of Mental Health and Well-Being. Psychol Med. 2003;33(2):357–62.

36. Kessler RC, Andrews G, Colpe LJ, Hiripi E, Mroczek DK, Normand SLT, et al. Short screening scales to monitor population prevalences and trends in non-specific psychological distress. Psychol Med. 2002;32(6):959–76.

37. Muthen L, Muthen B. Mplus Version 7 User's Guide: Version 7. Muthen & Muthen; 2012. 850 p.

38. Morin AJS, Arens AK, Tran A, Caci H. Exploring sources of construct-relevant multidimensionality in psychiatric measurement: A tutorial and illustration using the Composite Scale of Morningness. Int J Methods Psychiatr Res. 2016;25(4):277–88.

39. Morin AJS, Arens AK, Marsh HW. A Bifactor Exploratory Structural Equation Modeling Framework for the Identification of Distinct Sources of Construct-Relevant Psychometric Multidimensionality. Struct Equ Model Multidiscip J. 2016 Jan 2;23(1):116–39.

40. Asparouhov T, Muthén B, Morin AJS. Bayesian structural equation modeling with cross-loadings and residual covariances: Comments on Stromeyer et al. J Manag. 2015;41(6):1561–77.

41. Marsh HW, Hau K-T, Grayson D. Goodness of Fit in Structural Equation Models. In: Contemporary psychometrics: A festschrift for Roderick P McDonald.

For peer review only

551 Mahwah, NJ, US: Lawrence Erlbaum Associates Publishers; 2005. p. 275–340.
552 (Multivariate applications book series).

553 42. Hu L, Bentler PM. Cutoff criteria for fit indexes in covariance structure
554 analysis: Conventional criteria versus new alternatives. Struct Equ Model Multidiscip J.
555 1999 Jan 1;6(1):1–55.

556 43. Stucky BD, Edelen MO. Using hierarchical IRT models to create unidimensional
557 measures from multidimensional data. In: Handbook of item response theory modeling:
558 Applications to typical performance assessment. New York, NY, US: Routledge/Taylor &
559 Francis Group; 2015. p. 183–206. (Multivariate applications series).

560 44. Cohen J. Statistical power analysis for the behavioral sciences. 2nd ed. Hillsdale, N.J: L. Erlbaum
561 Associates; 1988. 567 p

562 45. R Foundation for Statistical Computing, Vienna, Austria. R Core Team. R: A
563 language and environment for statistical computing [Internet]. [cited 2019 Oct 21].
564 Available from: http://www.R-project.org/

565 46. Wege N, Li J, Muth T, Angerer P, Siegrist J. Student ERI: Psychometric
566 properties of a new brief measure of effort-reward imbalance among university students. J
567 Psychosom Res. 2017;94:64–7.

For peer review only

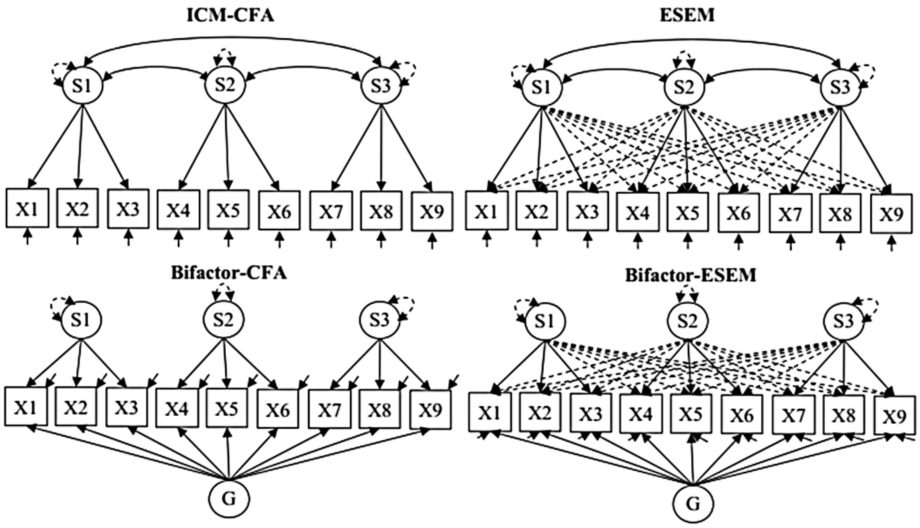

Figure 1. Simplified representations of specified models.

268x150mm (300 x 300 DPI)

STROBE Statement—checklist of items that should be included in reports of observational studies

	Item No.	Recommendation	Page No.	Lines No.
**Title and abstract**	1	(*a*) Indicate the study's design with a commonly used term in the title or the abstract<br>*The abstract describes the study as "a cross-sectional observational study".*	2	28-29
		(*b*) Provide in the abstract an informative and balanced summary of what was done and what was found.<br>*The abstract informs about the objectives and provides the results.*	2	28-45
**Introduction**				
Background/rationale	2	Explain the scientific background and rationale for the investigation being reported<br>*Background and rationale were provided in the Introduction, paragraphs 1, 2, 3, 4, 5, 6, 7, 8, 9.*	5-7	58-127
Objectives	3	State specific objectives, including any prespecified hypotheses<br>*Specific objectives were provided in the Introduction, paragraph 10.*	7	128-131
**Methods**				
Study design	4	Present key elements of study design early in the paper.<br>*The study design was discussed in the Methods section, in the "Participants and procedure" subsection, paragraphs 2, 3.*	8	141-156
Setting	5	Describe the setting, locations, and relevant dates, including periods of recruitment, exposure, follow-up, and data collection<br>*The setting and the location were described in the Methods section, paragraphs 1 & 2.*<br>*Relevant dates were described in the Methods section, in the "Participants and procedure" subsection, paragraph 3.*	8-9	135-156
Participants	6	(*a*) *Cross-sectional study*—Give the eligibility criteria, and the sources and methods of selection of participants<br>*Including criteria were reported in the Methods section, in the "Participants and procedure" subsection, paragraph 3.*	8	146-150
Variables	7	Clearly define all outcomes, exposures, predictors, potential confounders, and effect modifiers.<br>*All variables were listed in the Methods section, paragraph 3.*	9	155-156

For peer review only - http://bmjopen.bmj.com/site/about/guidelines.xhtml

Data sources/ measurement	8*	For each variable of interest, give sources of data and details of methods of assessment (measurement).<br>*Details of each measure were discussed in the Methods section, in the "measures" subsection, paragraph 1, 2, 3.*	*9-10*	*160-184*
Bias	9	Describe any efforts to address potential sources of bias<br>The attempt to avoid bias were mentioned in the Methods section, in the "Participants and procedure" subsection, paragraph 3.	8	151-153
Study size	10	Explain how the study size was arrived at<br>*The study size was described in the Methods section, in the "Participants and procedure" subsection, paragraph 2.*	8	141
Quantitative variables	11	Explain how quantitative variables were handled in the analyses. If applicable, describe which groupings were chosen and why<br>*Details regarding how each quantitative variable was handled was discussed in the Methods section, in the "Measures" subsection, paragraph 1, 2, 3.*	*9-10*	*160-184*
Statistical methods	12	(*a*) Describe all statistical methods, including those used to control for confounding<br>*All statistical methods are described and discussed in the "Statistical analyses" subsection.*	10-13	188-256
		(*b*) Describe any methods used to examine subgroups and interactions<br>*Not applicable.*	-	-
		(*c*) Explain how missing data were addressed<br>*Missing were not included in the study as stated in the Methods section, "Participants and procedure" subsection, paragraph 2.*	8	149-150
**Results**				
Participants	13*	(a) Report numbers of individuals at each stage of study—eg numbers potentially eligible, examined for eligibility, confirmed eligible, included in the study, completing follow-up, and analysed<br>*Not applicable.*	-	-
		(b) Give reasons for non-participation at each stage<br>*Not applicable.*	-	-
		(c) Consider use of a flow diagram<br>*Not applicable.*	-	-

For peer review only

Descriptive data	14*	(a) Give characteristics of study participants (eg demographic, clinical, social) and information on exposures and potential confounders<br>*Sociodemographics characteristics were described in the Results section, paragraph 1.*	13	259-262
		(b) Indicate number of participants with missing data for each variable of interest<br>*Not applicable.*		
Outcome data	15*	*Cross-sectional study*—Report numbers of outcome events or summary measures<br>*Not applicable.*		
Main results	16	(*a*) Give unadjusted estimates and, if applicable, confounder-adjusted estimates and their precision (eg, 95% confidence interval). Make clear which confounders were adjusted for and why they were included<br>*Not applicable.*		
		(*b*) Report category boundaries when continuous variables were categorized<br>*Not applicable.*		
		(*c*) If relevant, consider translating estimates of relative risk into absolute risk for a meaningful time period<br>*Not applicable.*		
Other analyses	17	Report other analyses done—eg analyses of subgroups and interactions, and sensitivity analyses<br>*Results were reported in the results section.*	13-18	258-339
**Discussion**				
Key results	18	Summarise key results with reference to study objectives<br>*The key results are discussed in the Discussion, paragraphs, 1, 2, 3, 4, 5, 6, 7.*	18-20	342-387
Limitations	19	Discuss limitations of the study, taking into account sources of potential bias or imprecision. Discuss both direction and magnitude of any potential bias<br>*The limitations of the study are described in the Discussion, paragraph 9.*	20	393-404
Interpretation	20	Give a cautious overall interpretation of results considering objectives, limitations, multiplicity of analyses, results from similar studies, and other relevant evidence<br>*A cautious overall interpretation of the results is given in the Discussion, paragraph 8.*	20	388-392
Generalizability	21	Discuss the generalizability (external validity) of the study results<br>*The representativeness of the sample is addressed in the Discussion, paragraph 9.*	20	393-397
**Other information**				

For peer review only - http://bmjopen.bmj.com/site/about/guidelines.xhtml

Funding	22	Give the source of funding and the role of the funders for the present study and, if applicable, for the original study on which the present article is based<br>*Information on the funding is given in the Funding section.*	21	420-421

*Give information separately for cases and controls in case-control studies and, if applicable, for exposed and unexposed groups in cohort and cross-sectional studies.

Note: An Explanation and Elaboration article discusses each checklist item and gives methodological background and published examples of transparent reporting. The STROBE checklist is best used in conjunction with this article (freely available on the Web sites of PLoS Medicine at http://www.plosmedicine.org/, Annals of Internal Medicine at http://www.annals.org/, and Epidemiology at http://www.epidem.com/). Information on the STROBE Initiative is available at www.strobe-statement.org.

For peer review only
